# Effects of Self-Perceived Oral Health and Stress Levels on Subjective Oral Symptoms and Lifestyle of University Students in South Korea: A Cross-Sectional Survey

**DOI:** 10.21203/rs.3.rs-4906078/v1

**Published:** 2024-09-18

**Authors:** YuYeon Jung, JinHyoung Jeong

**Affiliations:** 1 Department of Dental Hygiene, Catholic Kwandong University, Beomil-ro 579beon-gil, Gangneung-si, 25601, Gangwon-do, South Korea.; 2 Department of Biomedical Management, Catholic Kwandong University, Beomil-ro 579beon-gil, Gangneung-si, 25601, Gangwon-do, South Korea.

**Keywords:** Oral health, University students, Lifestyle, Stress, Self-perceived oral health

## Abstract

**Background:**

Self-perceived oral health is related to clinical and subjective oral factors, socioeconomic factors, perceived stress, and oral health behavior. However, limited studies have examined whether self-perceived oral health is related to dry mouth, salivary viscosity, or lifestyle factors. Accordingly, this study aimed to verify the effect of self-perceived oral health and stress levels on subjective oral symptoms and lifestyle.

**Methods:**

The responses of 644 university students who agreed to participate in the study were analyzed. A chi-square test was used to determine whether self-perceived oral health and stress levels showed significant differences based on subjective oral symptoms and lifestyle. Logistic regression was used to analyze the effect of subjective oral symptoms and periodontal disease diagnosis on self-perceived oral health.

**Results:**

Subjective oral symptom factors that showed significant differences depending on self-perceived oral health included gingival bleeding or pain, dry mouth, and saliva viscosity, and depending on lifestyle factors included the frequency of tooth brushing and beverage consumption. Additionally, subjective oral symptoms influenced self-perceived oral health. Self-perceived oral health was negative when there was gingival bleeding, pain (odds ratio (OR)=0.594, p=0.002), and dry mouth (OR=0.577, p=0.001).

**Conclusions:**

This study’s results showed that self-perceived oral health significantly impacts gingival bleeding, pain, and dry mouth. Therefore, government intervention must provide and manage innovative and efficient education programs that promote self-perceived oral health management habits and maintenance and improvement of oral health.

## 1 Background

Oral health is a multifaceted and essential component of good health, allowing us to perform daily activities, such as speaking, smiling, tasting, and chewing [1]. The World Health Organization defines oral health as freedom from conditions that limit a person’s psychosocial well-being capacity [2]. Maintaining oral health is crucial because it can improve mental and overall health [3]. Factors that determine oral health include physiological functions, psychosocial functions, disease states, environmental factors, and self-perceived oral health [4]. Self-perceived oral health is related to clinical and subjective oral factors, socioeconomic factors, perceived stress, and oral health behavior [5]. Specifically, self-perceived oral health is closely correlated with objective clinical indicators and is considered crucial to oral health because it provides insight into an individual’s oral health status [6]. Clinical factors include dental caries, tooth loss, and gingival bleeding; subjective factors are related to general health status and oral pain [5]. Additionally, clinical and subjective oral factors such as tooth loss, gingival bleeding, and pain influence perceptions of one’s oral health, well-being, and quality of life [5]. Therefore, to maintain self-perceived oral health, related factors must be controlled and managed.

Several countries are examining self-perceived oral health, including China and Japan [7, 8]. However, identifying the determinants of self-mouth health recognition is crucial because the factors associated with them vary by race [5]. Therefore, further studies on self-perceived oral health and overall oral health in South Korea are needed. Specifically, university students are young adults undergoing dynamic growth and may experience changes in their health, social psychology, lifestyle, and behavior [7]. Additionally, many university students are living away from home for the first time and are responsible for their health, lifestyle, and behavior; incorrect health behaviors can significantly impact their self-perceived oral health [5, 9]. Therefore, systematic research is needed to develop positive self-perceived oral health measures to ensure good oral health management among South Korean university students. Accordingly, this study aimed to investigate whether self-perceived oral health and stress are related to subjective oral symptoms and lifestyle among South Korean university students and suggest the importance of oral health management in university students.

## 2 Results

A chi-square test was conducted to verify whether there were significant differences in subjective oral symptoms and lifestyle factors according to the participants’ self-perception of oral health (Table 1). Among the participants, the number of those who perceived their oral cavity as healthy significantly exceeded the number of those who perceived it as unhealthy, and they reported no gingival bleeding or pain (66.4%), no dry mouth (66.0%), or meager saliva viscosity. (63.3%). Additionally, the proportion of participants who perceived their oral health as healthy was significantly higher when they brushed their teeth three times (64.0%) and when they did not consume any beverages (69.2%) than when they perceived their oral cavity as unhealthy. However, there was no statistically significant difference in self-perceived oral health between smoking status and sleep duration.

A chi-square test was conducted to verify whether there were significant differences in subjective oral symptoms and lifestyle factors according to participants’ stress levels (Table 2). Regarding subjective oral symptom factors, gingival bleeding or pain (55.1%), no dry mouth (55.6%), and very low salivary viscosity (54.3%) were observed when the stress level was low. Regarding lifestyle factors, when the stress level was low, the respondents were non-smokers (53.9%), and the average daily sleep duration was less than seven hours (59.3%). However, there was no significant difference in stress levels between the average daily tooth brushing frequency and average weekly frequency of beverage consumption.

**Fig. 1:**
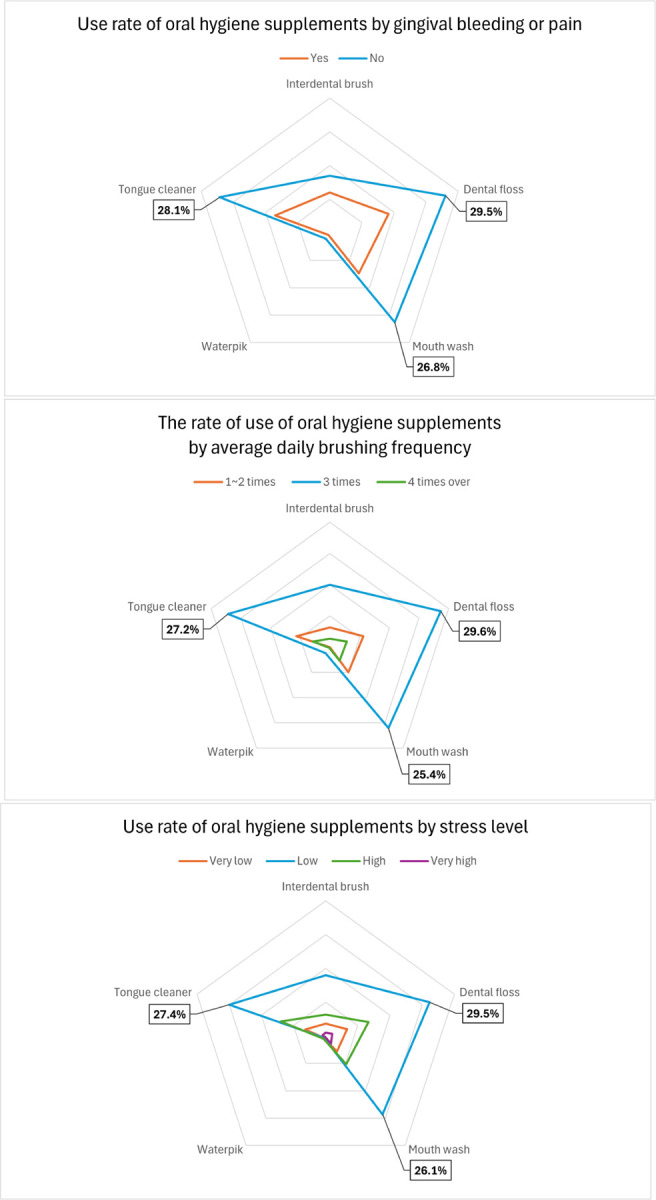
Factors influencing oral hygiene aid use: bleeding or pain, brushing frequency and stress level

Figure 1 shows the differences in the use of oral hygiene aids according to gingival bleeding or pain, average daily tooth brushing frequency, and stress levels among individuals who perceived their oral health as healthy. The rate of use of oral hygiene aids was highest when there was no gingival bleeding or pain, when the average daily tooth brushing frequency was three times, and when stress levels were low. Dental flosses, tongue cleaners, and mouthwashes were the most frequently used oral hygiene aids.

A chi-square test was conducted to determine the impact of gingival bleeding or pain, dry mouth, and a diagnosis of periodontal disease on self-perceived oral health (Table 3). When there was gingival bleeding or pain (odds ratio [OR]=0.594, p=0.002) or dry mouth (OR=0.577, p=0.001), the perception that one’s oral cavity was healthy was low. Therefore, subjective oral symptoms (gingival bleeding or pain and dry mouth) and self-perceived oral health may have a significant mutual influence.

## 5 Methods

### Study population

We used a random non-probability sampling technique for the survey. The research purpose and methods were explained through a self-administered structured questionnaire targeting university students, which was distributed through an online Google survey link. The survey responses of 644 people who voluntarily agreed were used for the final data analysis. This study was conducted following the Declaration of Helsinki.

### Questionnaire

The questionnaire comprised questions regarding general characteristics, self-perceived oral health, subjective oral symptoms, lifestyle, stress levels, and periodontal disease diagnosis. General characteristics included gender (male, female) and grade (1st, 2nd, 3rd, and 4th grades). Self-perceived oral health was classified as good and bad, and subjective oral symptoms were classified according to gingival bleeding or pain, the presence of dry mouth, and the viscosity of saliva (very low, low, high, very high). Lifestyle factors included the average frequency of daily tooth brushing (one to two times, three times, or four or more times), smoking status (yes/no), average weekly frequency of consumption of beverages such as soda and ade (no beverages consumed, one to two times, three to four times, or five or more times), average daily sleep duration (5 hours or less, 6 hours or less, 7 hours or less, or 8 hours or more), and oral hygiene aids used (not used, interdental brush, dental floss, mouthwash, water pick, or tongue cleaner). They were categorized by stress level (very low, low, high, or very high) and whether the respondent had been diagnosed with periodontal disease (yes/no).

### Statistical analysis

A chi-square test was conducted to determine whether there was a significant difference in participants’ self-perceived oral health and stress levels with their subjective oral symptoms and lifestyle, as well as the average daily oral health perception of those who considered their oral health healthy. The use of oral hygiene aids was analyzed according to tooth brushing frequency, gingival bleeding or pain, and stress level. Additionally, logistic regression analysis was conducted to verify the effect of subjective oral symptoms (gingival bleeding or pain and dry mouth) and the diagnosis of periodontal disease on self-perceived oral health. Statistical analyses were performed using SPSS Statistics ver. 22, and the significance level at the time of the decision was set at 0.05.

## 6 Discussion

Self-perceived oral health is used in epidemiological studies because, in addition to disease, it is influenced by functional capacity, pain, aesthetics, and psychosocial factors [10]. The key factors associated with self-perceived oral health include clinical and subjective oral health [5]. However, limited studies examine whether self-perceived oral health is related to dry mouth, salivary viscosity, or lifestyle factors. Investigating these factors is crucial to improve self-perceived oral health. Therefore, this study investigated factors related to self-perceived oral health among South Korean university students. The U.S. Centers for Disease Control and Prevention (CDC) defined oral health as the health status of teeth, gingiva, and oral facial systems, and reported that diseases affecting oral health include dental carries, periodontal disease, oral cancer, and tooth loss [11]. Periodontal disease is an inflammatory disease that affects the soft and hard tissues that support teeth and causes gingival bleeding, pain, redness, swelling, tooth agitation, and loss [12, 13]. When bleeding or pain occurs in the gingiva, it affects speech and pronunciation, causing difficulties in social interaction, and negatively affects psychological health through feelings of guilt, discomfort, or lack of care [14]. According to Luchi et al.’s study, people experiencing periodontal disease, dry mouth, cavities, and tooth loss often perceived their oral cavity as unhealthy [15]. This aligns with our results that people who perceived their oral health as healthy did not have gingival bleeding, pain, or dry mouth compared with people who perceived their oral health as unhealthy and had minimal saliva viscosity. Yins reported that self-perceived oral health is associated with the development of good oral health behaviors [16]. This aligns with our results, which showed that self-perceived oral health is significantly related to lifestyle. Lifestyle factors related to self-perceived oral health included average daily tooth brushing frequency and average weekly frequency of beverage consumption. The group that perceived their oral health as healthy brushed their teeth an average of three times per day (64.0%). People consider brushing their teeth a fundamental self-care behavior for maintaining oral health [17]. Tooth brushing helps prevent and relieve dental caries and dry mouth by removing plaque from teeth and temporarily increasing saliva secretion [18, 19]. Therefore, many people believe that brushing their teeth helps maintain overall physical hygiene and health, and they expect that brushing their teeth will make their mouths healthier [20]. This finding supports that self-perceived oral health is related to the frequency of tooth brushing. Moreover, self-perceived oral health was associated with beverage consumption habits. Specifically, Korean college students use energy drinks to overcome sleep deprivation when studying for exams or completing projects [21]. Energy drinks are used to improve mental and physical performance and have gained global popularity over the years, making them the fastest-growing drink in the global beverage market [22]. However, these drinks contain excess amounts of sugar and are highly acidic. Thus, they pose a danger to oral health due to the risk of leading to dental caries and tooth erosion. Furthermore, their excessive consumption can increase stress levels among university students with sleep disorders [23–25]. Therefore, their consumption has emerged as a global public health problem [22]. This shows how subjective oral symptoms and incorrect lifestyle habits significantly impact oral health, and the need for forming positive self-perceived oral health by practicing a healthy lifestyle. University students in South Korea are under considerable stress due to academics, pressure to succeed, competition, and financial burdens [26, 27]. University students under significant stress change their eating and lifestyle, negatively affecting their emotions and health [28, 29]. Therefore, we investigated and analyzed the relationships among stress, subjective oral symptoms, and lifestyle to determine the factors affecting university students’ health. Stress affects health directly and indirectly through changes in autonomic and neuroendocrine responses and health behaviors [30]. Specifically, if stress is not relieved, various problems arise. According to Stankeviciene’s study on stress among university students, high stress levels lead to unhealthy behaviors, such as incorrect eating habits, smoking, and drinking, leading to failure to control body homeostasis [28, 31]. Failure to maintain homeostasis causes weakening of the periodontal tissue and decreased saliva secretion due to local tissue destruction in the oral cavity [28, 32]. When saliva secretion decreases, complications such as dry mouth, dental caries, periodontal disease, oral burning, and oral pain occur [33–35]. Furthermore, they do not have antibacterial, mastication, digestion, taste, lubrication, or pH buffering effects, making it challenging to maintain oral homeostasis [36, 37]. Therefore, stress must be controlled appropriately to promote and maintain oral health. This study’s results showed that stress was closely related to subjective oral symptoms and lifestyle. Studies on the stress levels of university students show that when their stress level is low, they do not experience gingival bleeding, pain, or dry mouth, and the viscosity of their saliva is low. Stress also affects smoking and sleep duration. The correlation between stress and smoking has been investigated using various methods [38–40]. Cigarettes are perceived to calm the mind and lead to focused thoughts when stress levels are high [41]. Moreover, stress affects sleep duration and quality; the shorter the sleep duration, the more negatively the person reacts to daily stressors and events [42]. Blood pressure, heart rate, hormone secretion, immune function, cell repair, memory recovery, and cognitive function are all regulated during sleep [43]. Lack of sleep is also related to respiratory and cognitive function decline, memory loss, and weight gain and negatively impacts university students’ academic and overall health [43]. Therefore, the U.S. National Sleep Foundation recommends that adults get seven to nine hours of sleep [44]. People with high stress levels may neglect oral hygiene behaviors, leading to negative outcomes [45]. Additionally, studies show that stress increases anxiety, significantly impacting oral health [46]. Conversely, people with less anxiety may have better lifestyle-related and oral hygiene behaviors [46]. Oral hygiene behaviors include regular brushing, use of oral hygiene aids, and regular dental checkups; practicing these oral hygiene behaviors helps prevent dental caries and periodontal diseases [47]. Furthermore, it affected various factors, including gingival status, oral hygiene knowledge, and stress, aligning with this study [20, 48–50]. Therefore, this study investigated the effects of gingival bleeding or pain, average daily tooth brushing frequency, and stress levels on the use of oral hygiene aids according to self-perceived oral health. The results showed that the rate of use of oral hygiene aids was high when there was no gingival bleeding or pain, when the average daily brushing frequency was three times, and when the stress level was low, especially when the rate of use of oral hygiene aids was high, in the order of dental flossing, tongue cleaning, and gargling. Therefore, to maintain good oral health, it is critical to remove plaque daily, and dental floss or an interdental brush is recommended to reduce gingivitis and plaque compared with brushing alone [51–53]. Additionally, individuals who brush their teeth regularly use more oral hygiene aids. Consistent results have shown that people who perceived their oral health as healthy did not have gingival bleeding or pain and frequently used dental floss. A previous study reported that those who brushed their teeth more than three times per day were 1.394 times more likely to use interdental care products than those who brushed their teeth twice [54]. Additionally, people with knowledge of oral health are more likely to have better self-care habits [55]. Therefore, ongoing oral health education is required to increase the likelihood of people practicing better oral care habits [47]. Additionally, government intervention must provide and manage innovative and efficient educational programs to develop self-administered oral health management habits to maintain and improve oral health. Oral diseases are caused not only by children unfamiliar with dental care, but also by chronic diseases such as diabetes, high blood pressure, and osteoporosis; additionally, young and middle-aged people complain of periodontal diseases caused by stress [56]. Due to the chronic and cumulative nature of oral diseases, dental care expenses constitute a significant proportion of the total medical costs and continue to increase steadily each year; the 2020 results for common dental outpatient diseases indicated that “gingivitis and periodontal disease” affected 16.27 million people (31.4%), making it the most prevalent condition, followed by “dental caries,” which affected 6.13 million people (11.8%) [56]. Therefore, it is critical for university students to develop proper lifestyles as they begin adulthood [57, 58]; this study aimed to provide fundamental data for developing an efficient and practicable educational program for oral health management. However, because this study’s survey results are based on university students in South Korea, there are limitations to generalizing the results to all university students. Additionally, because this was a cross-sectional survey, causal relationships cannot be presented for mutual relationships. Future studies must analyze risk factors affecting health through systematic research targeting university students and explore various approaches to improve university students’ health and lifestyle and suggest the need for effective clinical implementation policies.

## 7 Conclusion

This study had several key findings. First, the correlations between self-perceived oral health and stress, subjective oral symptoms, and lifestyle factors were confirmed. Second, subjective oral symptoms, lifestyle, and stress were found to be related to the use of oral hygiene aids. Therefore, as both self-perceived oral health and stress harm oral health, it is crucial to establish a practical education program to improve lifestyle.

## Figures and Tables

**Table 1:** Association between subjective oral symptoms and lifestyle factors according to self-perceived oral health

Variable	N(%)		*χ*^2^(p)
	Self-perceived oral health		
	Good	Poor	
Gingival bleeding or pain			
Yes	138(52.5)	125(47.5)	120663 (<0.001) ***
No	253(66.4)	128(33.6)	
Dry mouth			
Yes	125(51.9)	116(48.1)	12.638 (<0.001) ***
No	266(66.0)	137(34.0)	
Saliva viscosity			
Very Low	253(63.3)	147(36.8)	8.638 (0.035)*
Low	92(61.3)	58(38.7)	
High	39(52.7)	355(47.3)	
Very high	7(35.0)	13(65.0)	
Daily tooth brushing frequency			
1~2	97(52.7)	87(47.3)	6.922 (0.031)*
3	258(64.0)	145(36.0)	
≥ 4	36(63.2)	21(36.8)	
Smoking			
Yes	50(56.2)	39(43.8)	0.890 (0.345)
No	341(61.4)	214(38.6)	
Average weekly beverage consumption frequency			
None	83(69.2)	37(30.8)	9.482 (0.024)*
1~2	194(62.8)	115(37.2)	
3~4	86(53.1)	76(46.9)	
≥ 5	28(52.8)	25(47.2)	
Average daily sleeping duration			
≤ 5*hr*	127(56.7)	97(43.3)	4.594 (0.204)
≤ 6*hr*	153(60.0)	102(40.0)	
≤ 7*hr*	80(67.8)	38(32.2)	
≥ 8*hr*	31(66.0)	16(34.0)	

Note.

* p <0.05;

*** p <0.001

**Table 2:** The relationship between subjective oral symptoms and lifestyle factors according to stress level

Variable	N(%)				*χ*^2^ (p)
	Stress				
	Very low	Low	High	Very high	
Gingival bleeding or pain					
Yes	21(8.0)	126(47.9)	97(36.9)	19(7.2)	15.130 (0.002) **
No	55(14.4)	210(55.1)	98(25.7)	18(4.7)	
Dry mouth					
Yes	25(10.4)	112(46.5)	86(35.7)	18(7.5)	8.771 (0.032)*
No	51(12.7)	224(55.6)	109(27.0)	19(4.7)	
Saliva viscosity					
Very low	43(10.8)	217(54.3)	123(30.8)	17(4.3)	25.939 (0.002) **
Low	20(13.3)	80(53.3)	43(28.7)	7(4.7)	
High	13(17.6)	31(41.9)	22(29.7)	8(10.8)	
Very high	0(0.0)	8(40.0)	7(35.0)	5(25.0)	
Daily tooth brushing frequency					
1~2	25(13.6)	87(47.3)	63(34.2)	9(4.9)	8.918 (0.178)
3	47(11.7)	218(54.1)	117(29.0)	21(5.2)	
≥4	4(7.0)	31(54.4)	15(26.3)	7(12.3)	
Smoking					
Yes	9(10.1)	37(41.6)	28(31.5)	15(16.9)	24.703 (<0.001) ***
No	67(12.1)	299(53.9)	167(30.1)	22(4.0)	
Average weekly beverage consumption frequency					
None	16(13.3)	61(50.8)	38(31.7)	5(4.2)	11.515 (0.242)
1~2	38(12.3)	172(55.7)	83(26.9)	16(5.2)	
3~4	17(10.5)	77(47.5)	59(36.4)	9(5.6)	
≥5	5(9.4)	26(49.1)	15(28.3)	7(13.2)	
Average sleeping duration					
≤5hr	21(9.4)	104(46.4)	80(35.7)	19(8.5)	18.297 (0.032)*
≤6hr	30(11.8)	136(53.3)	78(30.6)	11(4.3)	
≤7hr	17(14.4)	70(59.3)	24(20.3)	7(5.9)	
*geq*8hr	8(17.0)	26(55.3)	13(27.7)	0(0.0)	

Note.

* p <0.05;

** p <0.01;

*** p <0.001

**Table 3:** Adjustment of odds ratio and 95% confidence interval of predictors of subjective oral symptoms and periodontal disease diagnosis for self-perceived oral health.

Variable (ref.)	Self-perceived oral health (ref. Poor)		
	Odds ratio	95% CI	p-value
Gingival bleeding or pain (ref. No)	0.594	0.425–0.830	0.002**
Dry mouth (ref. No)	0.577	0.414–0.804	0.001**
Periodontal disease (ref. No)	0.811	0.529–1.241	0.334

Note.

** p <0.01;

CI. confidence interval

## Data Availability

The datasets used and/or analysed during the current study are available from the corresponding author on reasonable request.
